# T-cell-based diagnosis of tuberculosis infection in children in Lithuania: a country of high incidence despite a high coverage with bacille Calmette-Guerin vaccination

**DOI:** 10.1186/1471-2466-9-41

**Published:** 2009-08-18

**Authors:** Edita Hansted, Angele Andriuskeviciene, Raimundas Sakalauskas, Rimantas Kevalas, Brigita Sitkauskiene

**Affiliations:** 1 Department of Pediatric Diseases, Kaunas University of Medicine, Eiveniu 2, 50009 Kaunas, Lithuania; 2 Laboratory of Microbiology, Kaunas Medical University Hospital, Eiveniu 2, 50009 Kaunas, Lithuania; 3 Department of Pulmonology and Immunology, Kaunas University of Medicine, Eiveniu 2, 50009 Kaunas, Lithuania

## Abstract

**Background:**

Lithuania is a country with a high incidence of tuberculosis (TB), despite a high coverage with bacille Calmette-Guerin (BCG) vaccination. Until now the only method used to detect latent TB infection was the tuberculin skin test (TST). However, TST may have a cross reactivity to the BCG vaccine and to environmental mycobacteria. The aim of this study was to conduct assessments of the diagnostic accuracy of the T-cell based test (T SPOT TB) for TB in children who had previously been BCG vaccinated and compare these with the results of the TST.

**Methods:**

Between January 2005 and February 2007, children with bacteriologically confirmed TB, children having contacts with a case of infectious pulmonary TB and children without any known risk for TB were tested with both the TST and T SPOT TB.

**Results:**

The TST and T SPOT TB tests were positive for all patients in the „culture-confirmed TB“ group. Whereas, in the „high risk for TB“ group, the TST was positive for 60%, but the T SPOT TB test, only for 17.8%. Meanwhile the results for the „low risk for TB“ group were 65.4% and 9.6%, respectively. A correlation between the TST and T SPOT TB was obtained in the "culture-confirmed TB" group where the TST ≥15 mm (r = 0.35, p < 0.001).

**Conclusion:**

The T-cell based method is more objective than the TST for identifying latent TB infection in children who had been previously BCG vaccinated. This method could be useful in countries like Lithuania where there is a high incidence of TB despite a high coverage with BCG vaccination. It may also help to avoid unnecessary chemoprophylaxis when TST reactions are false-positive.

## Background

Rapid identification and adequate treatment of latent tuberculosis (TB) infection is a very important part of successful TB control [1]. Any person, especially a child, who is exposed to infectious pulmonary TB, has a high risk of getting a TB infection or disease [2]. Even in countries with a low prevalence of TB, 30-40% of the new cases are caused by the transmission of *Mycobacterium tuberculosis *from infectious cases [3,4].

Lithuania is a country of high incidence of TB despite a high coverage with bacille Calmette-Guerin (BCG) vaccination. In Lithuania the prevalence of TB doubled through 1990-1997; it has stabilized since 1998. The TB incidence rate ranges from 60-70/100,000 inhabitants [5,6]. Nevertheless, according to a national anti-TB drug resistance prevalence survey, an alarmingly high prevalence of drug-resistant TB is challenging Lithuania- by about 9% of new TB cases [6,7].

Since 1890, and until recently, the only method available for the detection of latent TB infection was the tuberculin skin test (TST). However, it has poor specificity, especially in the population vaccinated with BCG [2,4,8]. The hypothesis is that a repetition of the TST may induce a booster effect [9]. The TST may have a cross reactivity to the BCG vaccine and to environmental mycobacteria [4,8]. If a TST shows a false-positive reaction, the subject does not have TB. Nonetheless, potentially unnecessary treatment will be administered to that subject. It is known that an irrational treatment of active TB may be a factor predisposing anti-TB drug resistance [7]. Unnecessary chemoprophylaxis can induce a hepatotoxic effect.

Newly developed blood tests are based on the detection of interferon-gamma (INF-γ) secretion by T-cells in response to *Mycobacterium tuberculosis *antigens: early secreted antigenic target (ESAT-6) and culture filtrate proteins (CFP-10). These tests could constitute a more rapid, specific and sensitive method for detection of TB infection [2,4,8,10]. T-cell-based tests are not affected either by a prior BCG vaccination or by most environmental mycobacteria. Furthermore, they were recently integrated into certain guidelines of strategies for screening subjects exposed to TB [11,12]. The T SPOT TB is one of the new, T-cell-based tests for TB infection, the approved regulatory version of the *ex vivo *enzyme-linked immunospot assay. The aim of our study was to conduct assessments regarding the diagnostic accuracy of the T SPOT TB for TB in children in Lithuania and to compare these with the results from the TST in the same population.

## Methods

Between January 2005 and February 2007, patients (aged 10-17 years old) with TB, the subjects screened for having contact with a case of infectious pulmonary TB and the subjects without any known risk for TB who were seeking a prophylactic examination at the Department of Pediatric Diseases, Kaunas Medical University Hospital were prospectively invited to participate in this study. The study protocol was approved by the Regional Bioethics Committee of the Kaunas University of Medicine and written informed consent was obtained from all participants and/or their parents.

All study subjects were divided into the following three groups:

1) „Culture-confirmed TB“ group (n = 23) - these are subjects with a bacteriologically confirmed TB diagnosis.

2) „High risk for TB“ group (n = 45) - these are subjects living with a family member who is ill with infectious TB or having contact with such a person in their school-classes. The shortest time of exposure was 5 weeks. All subjects in this group were free of symptoms at the time of enrolment.

3) „Low risk for TB“ group (n = 52) - these are subjects with no identifiable risk for TB (no known history of contact with TB patient, no changes in X-ray nor any complaints).

All subjects were screened using chest radiography, a clinical examination and an interview regarding their history of exposure to TB. All of the studied subjects had been BCG vaccinated in the past (at their birth and 7 yrs) according to the Government's Health Programme recommendations of that time [13]. BCG status was determined by scar after vaccination. None of these subjects had received an anti-TB treatment in the past. None had symptoms of HIV infection nor were suspected of carrying HIV and all were from a population at a low risk for HIV.

Eight patients from the „culture-confirmed TB“ group who had provided consent were repeatedly tested with TST and T-SPOT. TB after 8 weeks of treatment with isoniasid, rifampicin, ethambutol and pyrazinamide in doses administered according to their weight.

### Tuberculin skin test (TST)

The TST was performed according to the Mantoux technique by using 2 units (bioequivalent to 5 units as per the international standard) of the purified protein derivative, PPD (Copenhagen Statens Serum Institute, Denmark). The transverse diameter of induration was measured after 72 hours by a well-trained staff. The TST reaction was considered positive when the induration was ≥10 mm [14].

### Determination of specific-INF-γ secreting T-cells

Before the TST testing 10 ml of a venous blood was drawn for the diagnostic test of *Mycobacterium tuberculosis*-specific IFN-γ secreting T-cells (T SPOT TB, Oxford Immunotec, Oxford, UK). The T SPOT TB test was performed using fresh blood (<5 hours at room temperature) according to the manufacturer's instructions. These tests were done in the laboratory of the Kaunas Medical University Hospital. The mononuclear cells from peripheral blood were seeded at 2.5 × 10^5 ^cells/well in single-well plates, and two separate pools of overlapping peptides spanning the full length of ESAT-6 and CFP-10 proteins were used together with the negative and positive controls. The individual spots were counted for ESAT-6 and CFP-10 by using manual counting with Trypan Blue and a haemocytometer. The responses were considered as positive when the number of spots in the test wells (against at least one of the two tested antigens) was ≥6, if the negative control had 0-5 spots, or ≥2× the number of spots in the negative control wells, if the negative control had 6-10 spots. This cutoff was predefined by the manufacturer's instructions and previous publications [15,16].

### Statistical analysis

The data were analyzed using the SPSS package for Windows 13. Descriptive statistics were used for rating single characteristics. The Student's T test was performed to determine significance levels. Comparisons between numbers of spots in the T SPOT TB test and the TST diameters of induration were performed using the non-parametric Mann-Whitney-Wilcoxon rank sum test and Kruskal-Wallis test for comparison of more than two groups. Kendall's rank correlation was used to measure the strength of dependence between two variables. The results are presented as the mean ± standard deviation (SD). The level was considered significant at p < 0.05.

## Results

One hundred and twenty subjects agreed to be included in this study. The characteristics of the studied subjects are presented in Table 1. The demographic characteristics of the subjects did not differ significantly. The BCG vaccination rate of the studied subjects was 100%. TST and T SPOT TB were performed to all the studied subjects under study thereby providing valuable results. The distribution of the TST and T SPOT TB results in the studied groups is presented in Table 2.

**Table 1:** Characteristics of studied subjects and results of TST and T SPOT TB test

		Groups		
Variables		Culture-confirmed TB	High risk for TB	Low risk for TB

Subjects n		23	45	52

	male	52.2	42.2	53.8
	
Sex, %	female	47.8	57.8	46.2

Age, yrs^†^		15.1 (2.3)	14.0 (1.7)	13.3 (2.3)

TST size (mm) ^†^		18.0 (3.4)*	8.7 (6.1)	10.0 (7.2)

T SPOT TB spot counts^†^		35.0 (22.5)*	3.0 (5.4)	2.9 (4.4)

^†^- data are presented as mean (SD)

*- p < 0.001 vs other groups

**Table 2:** Distribution of T SPOT TB and TST in studied groups

	Culture confirmed TB (n = 23)	High risk for TB (n = 45)	Low risk for TB (n = 52)
T SPOT TB (+) TST (+)	23*	7	3

T SPOT TB (+) TST (-)	0	1	2

T SPOT TB (-) TST (+)	0	20	31

T SPOT TB (-) TST (-)	0	17	16

-, negative; +, positive;

*- p < 0.001 vs other groups

All patients from the „culture-confirmed TB“ group showed a positive TST and had a positive T SPOT TB test indicating a sensitivity of 100%. In that group, 82.6% of subjects showed an induration ≥15 mm in the TST.

Comparatively, of the total 45 subjects in the „high risk for TB“ group, 27 (60%) showed a positive TST, but only 8 (17.8%) had a positive T SPOT TB test. Seven of the 8 T SPOT TB-positive subjects from the „high risk for TB“ group had TST with ≥10 mm induration, and one had TST with <10 mm induration. In the „low risk for TB“ group, the prevalence of positive TST and T SPOT TB tests was 65.4% and 9.6%, respectively (Tables 1 and 2). The prevalence of positive TST and positive T SPOT TB tests was significantly higher in the „culture-confirmed TB“ group. The mean size of the TST induration and the T SPOT TB spot counts also prevailed in this group, but did not differ significantly between the other two groups.

**Figure 1:**
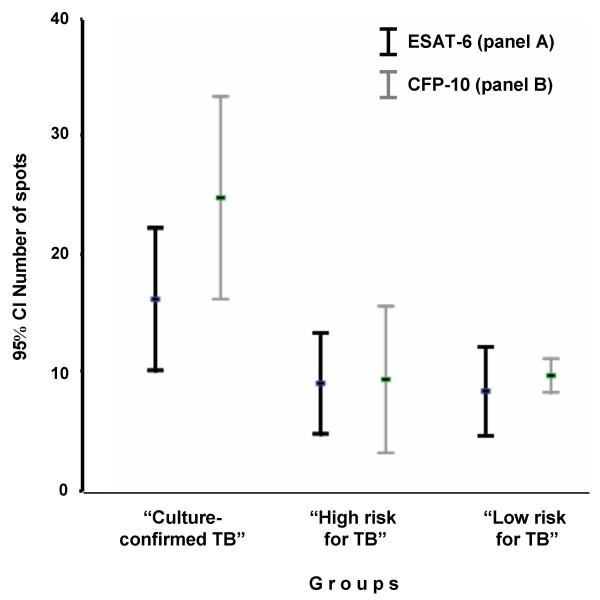
**Distribution of T SPOT TB results in relation to the studied groups and antigens used**. The comparison was performed considering only positive T SPOT TB results. The numbers of spots to ESAT-6 (T SPOT TB panel A) and CFP-10 (T SPOT TB panel B) in the „culture-confirmed TB“ group were significantly higher than in other groups (p < 0.001), but did not significantly differ in each group.

**Figure 2:**
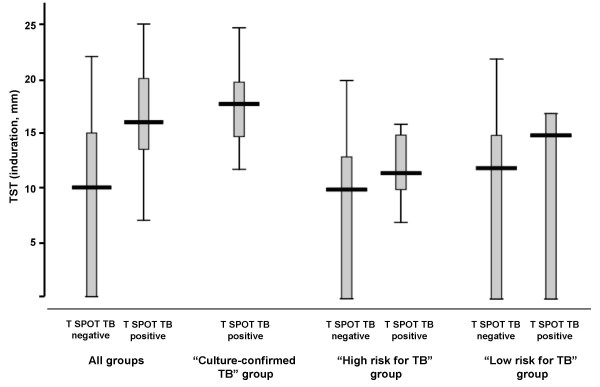
**Box and whisker plot of TST induration (in mm) in relation to the positive and negative T SPOT TB responses**. The boxes indicate the median, 25^th ^and 75^th ^quartiles, and minimum and maximum values.

The number of spots to ESAT-6 (T SPOT TB panel A) did not differ significantly from the spots count to CFP-10 (T SPOT TB panel B) in each group. However, they were significantly higher in the „culture-confirmed TB“ group than in the groups without clinical signs of TB (Figure 1). The results of TST (induration in mm) in relation to the positive and negative T SPOT TB responses are provided in Figure 2.

Subjects with both a positive TST and a positive T SPOT TB test (7 from the „high risk for TB“ group and 3 from the „low risk for TB“ group) were considered as being infected with *Mycobacterium tuberculosis*. One of the 18 TST-negative subjects from the „high risk for TB“ group and 2 of the 18 TST-negative subjects from the „low risk for TB“ group reacted positively in the T SPOT TB test. The reason of this discrepancy is unclear.

Because the cutoff for the TST is questionable, an attempt was made to analyze the T SPOT TB responses in relation to TST by applying different cutoffs. Depending on the TST cutoff, the studied subjects were divided into the following four groups: TST <5 mm, TST ≥5 and <10 mm, TST ≥10 and <15 mm, and TST ≥15 mm. The numbers of positive T SOPT TB subjects based on the four different TST cutoff values are presented in Table 3. A significant correlation between the TST and T SPOT TB tests was obtained in the "culture-confirmed TB" group where TST ≥15 mm (r = 0.35, p < 0.001).

**Table 3:** The numbers of positive T SOPT TB subjects based on the four different TST cutoff values

No. of persons	TST <5 mm	TST 5-9 mm	TST 10-14 mm	TST ≥15 mm
Culture-confirmed TB	0/0	0/0	4/4	19/19

High risk for TB	0/12	1/6	4/16	3/11

Low risk for TB	2/16	0/2	0/13	3/21

Data are presented as n (of subjects with a positive T SPOT TB)/n (of subjects with defined TST cutoff value)

**Figure 3:**
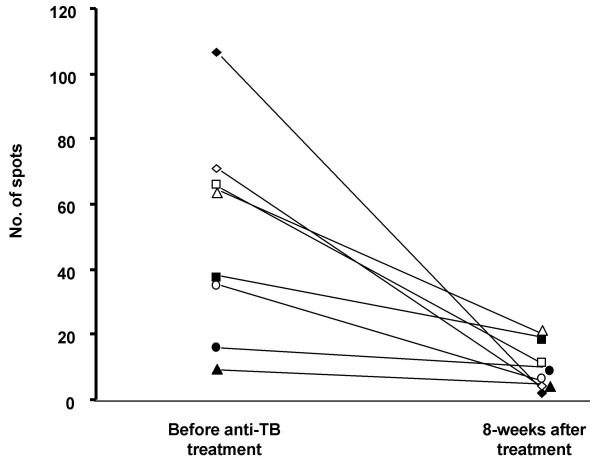
**Change of T SPOT TB results after 8-weeks anti-tuberculosis (TB) therapy in culture-confirmed TB subjects**. Each symbol represents an individual patient.

Furthermore, an investigation of the influence of anti-TB treatment on TST and T SPOT TB test results was conducted for this study. Eight children from the "culture-confirmed TB" group were repeatedly investigated after 8 weeks of anti-TB treatment. The TST results were still positive after the treatment (TST induration 17.9 ± 3.9 mm before treatment and 16.0 ± 3.5 mm after treatment). However, the number of T SPOT TB spot counts was reduced significantly after the treatment (no. of spots 50.3 ± 11.2 vs 9.8 ± 2.3 respectively, p = 0.01) (Figure 3). After 8 weeks of the anti-TB treatment, positive T SPOT TB results reverted in 5/8 children from the "culture-confirmed TB" group; whereas, in 3 children (with proven multidrug-resistant TB) the T SPOT TB test was still positive.

## Discussion

This study reports the results of the first evaluation of *ex vivo *INF-γ-based assay (T SPOT TB) for the diagnosis of TB infection in children in a country of a high incidence of TB despite a high coverage with BCG vaccination, and presents the comparison of T SPOT TB results with those of the TST. In this study we found that 100% of the children with a bacteriologically confirmed TB diagnosis had both a positive T SPOT TB and TST. However, the prevalence of positive TST in the studied children at a "high risk for TB" and in the children at a "low risk for TB" was significantly higher than the prevalence of positive T SPOT TB results was in the same groups. A striking observation was that 70% of the studied children from all three groups had a positive TST and only 39% of children with a positive TST had a positive T SPOT TB.

There is no gold standard for the diagnosis of latent TB infection [17,18]. TST is routinely used as the first step in the diagnosis of TB infection. According to the results of this study, the prevalence of positive TST is high, even in the "low risk for TB" group. TST may be false-positive in many cases of studied children, since the coverage of BCG vaccination in our country is high. All the studied children had a BCG vaccination in early childhood (at their birth and 7 yrs). Previous studies have shown that the TST does not accurately reflect the risk of latent TB infection in a setting with extensive BCG coverage [12,19,20]. There is a hypothesis that repetition of TST may induce a booster effect [9]. The specificity of TST may be not adequate due to cross-reactions not only with BCG vaccination, but also with non-tuberculous mycobacteria which appears to be rising, especially among young children [21]. It is important to note that false-positive TST reactions may lead to unnecessary treatment [16,22]. Chemoprophylaxis of TB infected persons is an important component of TB control strategy; however, due to the significant risk of hepatotoxicity and other side effects of anti-TB treatment, chemoprophylaxis cannot be prescribed only on the basis of the TST [8,10,23], especially in children.

In this study, all the children with a bacteriologically confirmed TB diagnosis had a positive T SPOT TB test. Other studies using the same assay system also demonstrated sensitivity values of 90% and 100% [15,24]. Various studies have shown that the T SPOT TB is more, rather than less, sensitive test than is the TST [2,8]. Moreover, the extremely high correlation of the TST with the BCG vaccination showed that false-positive reactions to the TST are rather than false-negative reactions to the T SPOT TB test [8]. However, there are also studies demonstrating the discordant results. Some of the studies, mainly performed with the other T-cell-based test QuantiFERON-TB (QFT), showed a fairly high level of negative results even among patients with active TB [25]. Antigens ESAT-6 and CFP-10 are specific for *Mycobacterium tuberculosis*, but they do not represent all antigenicity of *Mycobacterium tuberculosis *[26]. Lighter et al. studied 207 children with the TST and QFT, and found an excellent correlation between the negative TST and negative QFT results; however, only 23% of the studied children with a positive TST had a positive QFT [27]. Kang et al. found the positive QFT rates 4%, 10%, 44% and 81% in low risk, casual contact, close contact, and active TB patients, respectively [25]. Whereas in our study, the positive T SPOT TB results were: 9.6%, 17.8% and 100% in low risk, high risk for TB and bacteriologically confirmed TB groups. Such discrepancy between the results could be explained by the different distribution of patients to the groups (we did not differentiate TB-contacts), also by the different status of BCG vaccination. In our study, 100% of studied children showed a BCG scar, whereas in the Korean study only 67% from TB-close contacts had such scar; that is why they could be more susceptible for TB infection.

Despite affirmation by the subjects from the "low risk for TB" group that they had no known contact with a TB-sick person, 3 of them showed positive TST and T SPOT TB test. This fact caused a question about some unknown exposure to infectious pulmonary TB. There is a possible limitation to this present study that should be mentioned. The subjects enrolled into the study were actively seeking a prophylactic examination; they were not part of a randomly selected population. All subjects without clinical signs of TB, but with both positive TST and T SPOT TB tests were considered as having latent TB infection. This definition was arbitrary. Indeed, as mentioned before, there is no gold standard for defining latent TB infection [17,18].

Three from studied children with a negative TST test showed a positive T SPOT TB test. The reason of that is unclear. Children with a negative TST and a positive T SPOT TB may have a false-negative TST due to an intercurrent viral infection. Or, the possible explanation of this discrepancy would be that the T SPOT TB results were false positive or that the subjects were truly infected with *Mycobacteria tuberculosis *or another ESAT-6 or CFP-10 containing non-TB mycobacteria [8,19]. Franken et al. reported that 41% of the studied subjects with negative TST results in association with positive T SPOT TB assay (mainly to CFP-10) one year later had converted to positive TST results in association with negative T SPOT TB results [28]. In previous studies, it has been postulated that the response to the CFP-10 antigen might be indicative of active replication of the bacteria; whereas, the responses to the ESAT-6 antigen may persist as an immunological "scar" [28,29]. Further studies are needed to gain a better understanding of this phenomenon. In this study, the number of spots to ESAT-6 did not significantly differ from the count of spots to CFP-10. However, in children with confirmed-TB, the ESAT-6 and CFP-10 were significantly higher than they were in the TST-positive or TST-negative children from the other groups.

Furthermore, we have also analyzed the T SPOT TB responses in relation to TST using different cutoffs, because a TST cutoff ≥5 mm for contacts who have been previously BCG vaccinated is questionable [3,16]. All children with culture-confirmed-TB showed a TST cutoff ≥10 mm. Agreement between the TST and T SPOT TB results was obtained only in children with culture-confirmed-TB when the TST was ≥15 mm, but the discordance between these tests in other children shows that they are not equivalent. As many as 51 of the 61 individuals (83.6%) from the "high risk for TB" and "low risk for TB" groups with a TST reaction ≥10 mm were T SPOT TB negative. Data from this and previous studies confirm the lower sensitivity of TST compared to the INF-γ assay [16,22,30,31]. Even with an increased cut-off for the TST of 15 mm induration, 26 of the children from these groups would still have been indicated for chemoprophylaxis though their T SPOT TB tests were negative. Chee et al. also analysed levels of agreement between the TST and the INF-γ assay, but using a 15 mm induration cutoff for the TST did not make a substantial difference to the test results [29].

During the 8-weeks anti-TB treatment of children with culture-confirmed TB, the number of spots in the T SPOT TB test declined markedly; whereas, anti-TB treatment did not influence the TST results. Three children (from 8) treated with anti-TB drugs were still positive in their T SPOT TB tests after treatment. The reason why these children still had a high degree of positivity in their T SPOT TB tests may be linked with the persistence of a high bacteriological load as they had the multidrug-resistant form of TB. In previous studies, it was also shown that, in patients responding to therapy, the number of TB-specific T cells can decline below the cutoff value after 3 months [4,15,32]. All these data permits a suggestion that the INF-γ assay could be useful for monitoring the efficacy of TB therapy.

The data of this study show that the INF-γ-based method (like the T SPOT TB) is more objective and accurate than the TST for identifying latent TB infection in children. Since in our country with high coverage of BCG vaccination, where TST is currently the only tool routinely used for detecting latent TB infection, the false-positive TST reactions may lead to potentially unnecessary preventive treatment. This also may lead to incorrect statistical data regarding latent TB cases. The INF-γ-based assay could be a very useful addition to the diagnostic algorithm for children with suspected TB and may help to avoid unnecessary chemoprophylaxis.

## Conclusion

The T-cell-based method is more objective than the TST for identifying latent TB infection in children, especially in countries like Lithuania which have a high incidence of TB despite a high coverage with BCG vaccination. This may also help to avoid unnecessary chemoprophylaxis when TST reactions are false-positive.

## Abbreviations

BCG: bacille Calmette-Guerin; CFP-10: culture filtrate protein 10; ESAT-6: early secreted antigenic target 6; IL: interleukin; INF-γ: interferon-gamma; QFT: QuantiFERON-TB; TB: tuberculosis; TST: tuberculin skin test

## Competing interests

The authors declare that they have no competing interests.

## Authors' contributions

EH carried out screening, clinical evaluation and the part of T-cell-based diagnostic tests, and participated in the writing of the manuscript. AA carried out the major part of T-cell-based diagnostic tests. RS participated in the study design and in the sequence alignment, and participated in the writing of the manuscript. RK participated in the study design and in the sequence alignment. BS conceived the study, participated in its design, and participated in the writing of the manuscript. All authors read and approved the final manuscript.

## Pre-publication history

The pre-publication history for this paper can be accessed here:


